# A Critical Reassessment of Inflammatory and Nutritional Indices (PNI, GNRI, SII, NLR, PLR) for Predicting Arteriovenous Fistula Maturation and Long-Term Patency: A 945-Patient Cohort

**DOI:** 10.3390/jcm15124543

**Published:** 2026-06-11

**Authors:** Mehmet Aslan, Burak Duman, Umut Serhat Sanrı, Oğuz Karahan

**Affiliations:** 1 Department of Cardiovascular Surgery, Alanya Education and Research Hospital, 07400 Antalya, Turkey; 2 Department of Cardiovascular Surgery, Faculty of Medicine, Alaaddin Keykubat University, 07400 Antalya, Turkeyoguzk2002@gmail.com (O.K.)

**Keywords:** arteriovenous fistula, hemodialysis, vascular patency, biomarkers, systemic inflammation, nutritional status

## Abstract

**Background**: While recent literature emphasizes the predictive value of composite inflammatory and nutritional indices for vascular outcomes, this study evaluates the actual predictive capacity of preoperative indices (PNI, GNRI, SII, NLR, PLR) for de novo arteriovenous fistula (AVF) maturation and 1-year primary patency. **Methods**: We retrospectively analyzed 945 end-stage renal disease patients who underwent strictly radio-cephalic autologous AVF creation. Preoperative indices were calculated from routine parameters. Diagnostic accuracy for predicting 1-year patency loss was assessed using receiver operating characteristic (ROC) curves, and a multivariate logistic regression model was constructed to adjust for baseline anatomical and clinical variables. Targeted subgroup analyses evaluated high-risk populations, including those with diabetes, coronary, and peripheral artery disease. **Results**: The 1-year primary and secondary patency rates were 73.3% and 93.1%, respectively. In contrast to prevalent reports, no significant differences in preoperative PNI, GNRI, NLR, PLR, or SII scores existed between patients with patent and thrombosed fistulas (*p* > 0.05). ROC analyses showed no predictive utility (AUC: 0.476–0.518). Crucially, multivariate logistic regression revealed that preoperative arterial (OR: 0.58, *p* < 0.001) and venous diameters (OR: 0.51, *p* < 0.001) were the strongest independent predictors of AVF failure, whereas all systemic biomarkers lacked independent predictive significance. Subgroup analyses confirmed these indices failed to predict AVF outcomes even in high-risk settings with severe endothelial dysfunction. **Conclusions**: Preoperative composite nutritional and inflammatory indices do not independently predict AVF maturation or long-term patency when adjusted for local anatomy. Local anatomical features and hemodynamics heavily dominate vascular outcomes, indicating that systemic biomarkers have limited standalone clinical utility for guiding preoperative vascular access planning.

## 1. Introduction

Autologous arteriovenous fistulas (AVFs) remain the undisputed “gold standard” for vascular access in patients with end-stage renal disease (ESRD) requiring long-term hemodialysis due to their superior longevity and lower complication rates compared to synthetic grafts and central venous catheters [1]. Even with meticulous preoperative mapping and flawless surgical technique, up to 20–50% of newly created fistulas cannot be utilized due to primary maturation failure or early thrombosis [2,3]. The primary etiology of AVF failure is neointimal hyperplasia, driven largely by hemodynamic shear stress, surgical trauma, and severe endothelial dysfunction.

In recent years, the pathophysiological understanding of chronic kidney disease (CKD) has evolved beyond simple renal failure into a systemic state of accelerated premature aging, chronic oxidative stress, and profound immune dysregulation [4,5]. In the ESRD population, this systemic biological degradation is often characterized by the Malnutrition–Inflammation–Atherosclerosis (MIA) syndrome [6]. Due to this pathological landscape, nephrologists and surgeons have actively sought preoperative biomarkers capable of identifying patients at high risk for AVF failure.

Current research has extensively evaluated the use of next-generation systemic inflammatory and nutritional indices, such as the Neutrophil-to-Lymphocyte Ratio (NLR), Platelet-to-Lymphocyte Ratio (PLR), Systemic Immune-Inflammation Index (SII), Prognostic Nutritional Index (PNI), and Geriatric Nutritional Risk Index (GNRI) [7,8,9,10,11]. The increasing clinical interest in utilizing these systemic peripheral markers to predict anatomical and surgical outcomes necessitates rigorous clinical inquiry. While composite indices like PNI and GNRI may accurately reflect a patient’s overall cardiovascular mortality or general frailty profile [12,13], their specific diagnostic validity and independent predictive performance regarding the localized neointimal hyperplasia of a de novo surgical anastomosis warrant further comprehensive evaluation, particularly when local anatomical factors are considered.

The primary objective of this study is to critically reassess whether preoperatively calculated composite indices truly and independently predict AVF maturation and 1-year primary patency in a large, real-world hemodialysis cohort. By evaluating a strictly homogeneous cohort of 945 patients undergoing primary radio-cephalic AVF creation, integrating these biomarkers with local anatomical variables in a multivariate framework, and performing targeted subgroup analyses in high-risk populations (Diabetes Mellitus, Coronary Artery Disease, and Peripheral Artery Disease), this study aims to recalibrate current perspectives and clarify the actual standalone clinical utility of these systemic biomarkers in preoperative vascular surgical planning.

## 2. Materials and Methods

### 2.1. Study Design and Population

**Figure 1:**
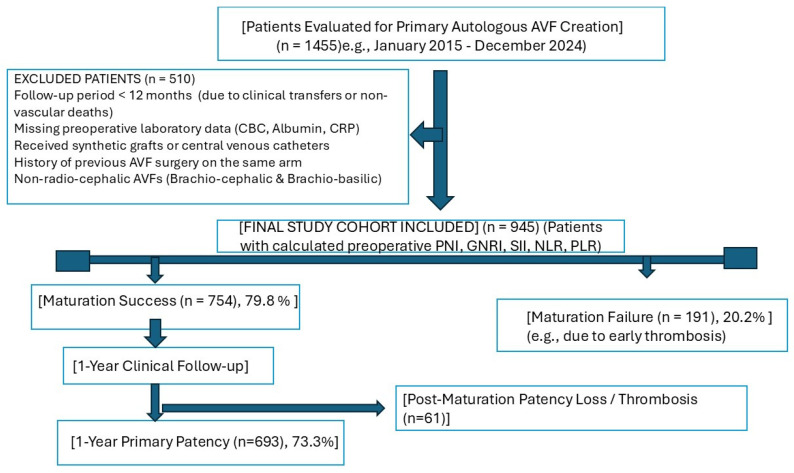
Graphical abstract summarizing the overall study design and main findings. The predictive effects of preoperative composite inflammatory and nutritional blood indices (NLR, PLR, SII, PNI, GNRI) on de novo arteriovenous fistula (AVF) maturation and 1-year primary patency were investigated in a cohort of 945 patients with end-stage renal disease (ESRD). The schematic highlights that the predictive values of all tested indices lacked independent clinical significance when adjusted for local anatomical parameters, failing to reliably predict localized AVF thrombosis in clinical practice.

This study was designed as a retrospective, observational cohort investigation to critically reassess the prognostic validity of popular composite biomarkers. To ensure a strictly homogeneous anatomical baseline and standardize the opportunity for repeated proximal interventions, only patients who underwent primary radio-cephalic (RC-AVF) creation were included. Brachio-cephalic and brachio-basilic fistulas were excluded. A total of 945 patients diagnosed with ESRD who underwent their first autologous RC-AVF creation were included in the study (Figure 1). Demographic, clinical, and operative data were obtained from the hospital information management system using a standardized data collection form. Preoperative Doppler ultrasonography (USG) measurements of arterial and venous diameters, along with comorbidities—Diabetes Mellitus (DM), Hypertension, Coronary Artery Disease (CAD), and Peripheral Artery Disease (PAD)—were recorded in detail to facilitate high-risk subgroup analyses. Routine laboratory data (Complete Blood Count, Albumin, CRP) obtained within the last month prior to surgery were integrated into the dataset.

Patients who died during the follow-up period were deliberately excluded to prevent confounding from non-vascular mortality (competing risks) and follow-up attrition. Importantly, patients whose fistulas thrombosed or failed within the first 12 months were strictly retained in the dataset and classified into the ‘failed’ cohort, thereby categorically preventing survivorship bias. The “follow-up period < 12 months” exclusion criterion exclusively referred to patients who were transferred to other centers or died.

### 2.2. Calculation of Composite Indices

Using preoperative blood counts and biochemical parameters, composite indices reflecting systemic inflammatory and nutritional status were calculated according to established formulas: NLR: Neutrophil/Lymphocyte, PLR: Platelet/Lymphocyte, SII: (Platelet × Neutrophil)/Lymphocyte, PNI: 10 × Albumin (g/dL) + 0.005 × Lymphocyte (Absolute), GNRI: 14.89 × Albumin (g/dL) + 41.7 × (Current Weight/Ideal Weight).

### 2.3. Endpoints and Statistical Analysis

The primary endpoints of the study were maturation success and the 1-year primary patency rate. AVF maturation was defined clinically as successful, complication-free cannulation with two needles for three consecutive dialysis sessions, or the fulfillment of the ‘Rule of 6s’ criteria upon Doppler ultrasonography evaluated at 4–6 weeks postoperatively. Secondary patency was defined as the re-establishment of functional dialysis flow following early or late thrombosis via endovascular interventions (e.g., balloon angioplasty) or open surgical revisions. Continuous variables were expressed as mean ± standard deviation (SD) and compared using independent samples *t*-tests. Categorical variables were expressed as numbers (*n*) and percentages (%). Receiver Operating Characteristic (ROC) curve analyses were performed to evaluate the diagnostic and predictive accuracy of composite indices for AVF failure, and the Area Under the Curve (AUC) was calculated. One-year cumulative primary patency durations for patients classified as high and low risk based on specific cut-off values (e.g., SII ≥ 1628) were evaluated using Kaplan–Meier survival analysis, and the Log-rank test was used for statistical comparison of survival differences between groups. Furthermore, specific subgroup analyses were conducted solely for patients with Diabetes Mellitus, Coronary Artery Disease, and Peripheral Artery Disease to determine if these biomarkers hold predictive value in pathologically severe conditions.

To rigorously evaluate the independent prognostic value of these biomarkers, a multivariate logistic regression model was constructed. Established determinants of AVF outcomes, including patient age, gender, Diabetes Mellitus, and precise preoperative arterial and venous diameters, were included in the model alongside the systemic indices to identify independent predictors of 1-year AVF failure. The level of statistical significance was set at *p* < 0.05.

## 3. Results

### 3.1. Demographic Characteristics, Clinical, and Laboratory Data

The mean age of the 945 patients included in the study was 61.5 ± 11.6 years, and 55.0% of the cohort consisted of male patients. The most common comorbidities within the cohort were Hypertension (79.0%), Diabetes Mellitus (44.7%), Coronary Artery Disease (33.4%), and Peripheral Artery Disease (16.5%). In preoperative Doppler USG evaluations, the mean arterial diameter of the target vessels was measured as 3.24 ± 0.90 mm, and the mean venous diameter was 3.62 ± 1.04 mm. Regarding surgical anatomy preferences, 100.0% of the AVFs analyzed in this homogeneous cohort were radio-cephalic (Table 1), as other configurations were excluded to maintain an exact anatomical baseline.

### 3.2. Clinical Outcomes

In the clinical course, the maturation success rate during the initial evaluation period was 79.8%, and the overall one-year primary patency rate was determined to be 73.3% (693 patent, 252 failed/thrombosed). The one-year secondary patency rate was 93.1% (*n* = 880). This robust secondary patency rate reflects the prompt angiographic and surgical salvage procedures facilitated by the continuous follow-up capacity of our clinic (Table 2).

### 3.3. Lack of Predictive Value in the Overall Cohort

**Figure 2:**
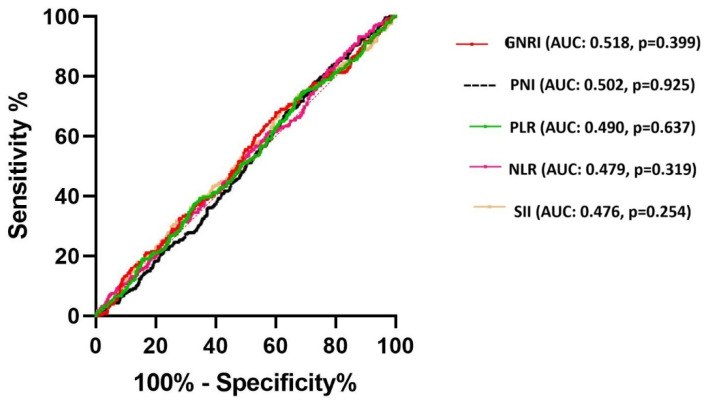
Combined Receiver Operating Characteristic (ROC) curve analysis of five different preoperative composite biomarkers (SII, NLR, PLR, PNI, GNRI) utilized to predict arteriovenous fistula (AVF) failure. It is clearly observed that the Area Under the Curve (AUC) values for all indices tightly cluster around the 0.50 reference line (identity line), indicating that none of the markers possess a significant independent predictive value.

**Figure 3:**
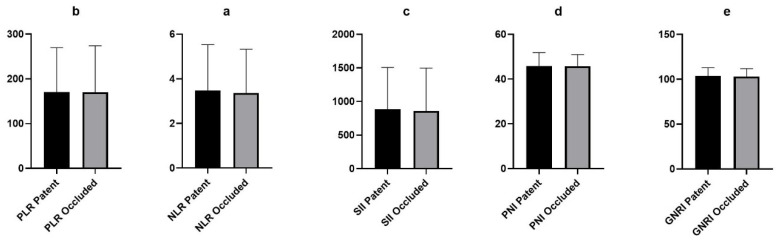
Comparison of preoperative composite inflammatory and nutritional indices between patent and occluded (failed) arteriovenous fistula groups. Bar graphs represent the mean values with standard deviations for (**a**) Neutrophil-to-Lymphocyte Ratio (NLR), (**b**) Platelet-to-Lymphocyte Ratio (PLR), (**c**) Systemic Immune-Inflammation Index (SII), (**d**) Prognostic Nutritional Index (PNI), and (**e**) Geriatric Nutritional Risk Index (GNRI). Consistent with the overall findings, no statistically significant differences were observed between the groups for any of the tested biomarkers (all *p* > 0.05).

Contrary to prevailing hypotheses in recent literature, our analysis revealed no statistically significant differences in preoperative inflammatory or nutritional composite scores between patients who maintained AVF patency and those who experienced a 1-year loss of patency. Specifically, the mean Systemic Immune-Inflammation Index (SII) was 885.00 in the patent group compared to 861.37 in the failed group (*p* = 0.412). Similar non-significant trends were observed for NLR (3.48 vs. 3.37; *p* = 0.355), PLR (170.40 vs. 169.79; *p* = 0.824), PNI (45.85 vs. 45.74; *p* = 0.751), and GNRI (103.73 vs. 103.00; *p* = 0.688) (Table 3, Figure 2 and Figure 3).

**Figure 4:**
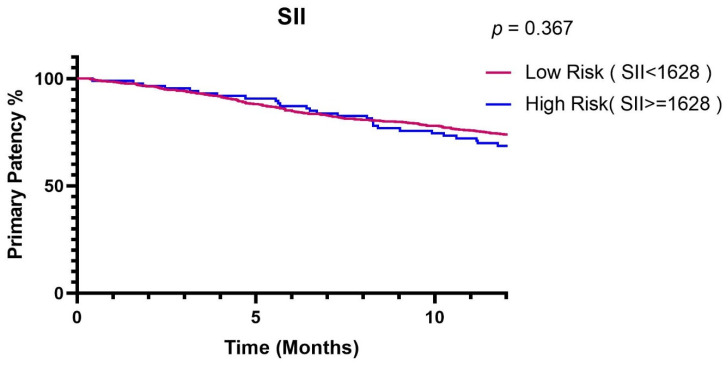
Kaplan–Meier survival estimate comparing 1-year primary patency rates stratified by preoperative Systemic Immune-Inflammation Index (SII) levels. The cohort was divided into high-risk (SII ≥ 1628) and low-risk (SII < 1628) groups. There was no statistically significant difference in cumulative patency survival between the two groups (Log-rank test, *p* = 0.367), with the survival curves completely overlapping. This strictly confirms that the patient’s systemic inflammatory burden fails to independently predict localized early vascular access failure within our cohort.

Consequently, ROC curve analyses demonstrated a profound lack of diagnostic accuracy for all tested indices in predicting AVF patency loss. Area Under the Curve (AUC) values ranged from 0.476 to 0.518, indicating that the predictive capacity of these preoperative biomarkers in our large cohort was not clinically significant (Table 4). Furthermore, Kaplan–Meier analysis revealed no statistically significant difference in 1-year cumulative primary patency rates between high-risk (SII ≥ 1628) and low-risk (SII < 1628) groups established based on a specific cut-off value (Log-rank test, *p* = 0.367) (Figure 4).

### 3.4. Failure of Biomarkers in High-Risk Subgroups

To further investigate whether these indices hold predictive value in patients with severe pre-existing endothelial dysfunction and advanced atherosclerosis, isolated subgroup analyses were performed for patients with Diabetes Mellitus (*n* = 422), Coronary Artery Disease (*n* = 316), and Peripheral Artery Disease (*n* = 156).

Strikingly, the biomarkers consistently failed to predict AVF outcomes even in these high-risk environments. Among diabetic patients, there was no significant difference in SII (1006.87 patent vs. 944.85 failed; *p* = 0.40), NLR (*p* = 0.31), or GNRI (*p* = 0.60) scores. Although PNI showed a borderline trend (*p* = 0.063), it paradoxically presented higher values in the failed group (45.51) compared to the patent group (44.43), effectively refuting the assumption that a lower nutritional score accelerates failure. Similar statistical insignificance was documented in the CAD cohort (*p* > 0.60 for all indices) and the PAD cohort (*p* > 0.14 for all indices), definitively demonstrating that systemic inflammatory and nutritional indices do not correlate with localized AVF survival, regardless of the patient’s baseline cardiovascular risk profile (Table 5).

### 3.5. Multivariate Predictors of AVF Failure

To rule out potential confounding and definitively test the independent prognostic value of these indices relative to established risk factors, a multivariate logistic regression model was constructed. Variables incorporated into the model included patient age, gender, diabetes mellitus, precise preoperative arterial and venous diameters, and prominent systemic indices (SII, NLR, and PNI). The analysis confirmed that local anatomical features heavily dominate vascular outcomes. Specifically, each 1 mm increase in preoperative arterial diameter (Odds Ratio [OR]: 0.58, 95% CI: 0.46–0.73, *p* < 0.001) and venous diameter (OR: 0.51, 95% CI: 0.41–0.63, *p* < 0.001) significantly reduced the risk of 1-year AVF failure. Conversely, none of the systemic inflammatory or nutritional biomarkers demonstrated any independent predictive value when adjusted for local anatomy and baseline demographics (Table 6).

## 4. Discussion

This large-scale cohort study critically reassessed the clinical utility of next-generation composite blood indices (PNI, GNRI, NLR, PLR, SII) in predicting vascular outcomes in hemodialysis patients. Our data clearly demonstrate that in a large and anatomically standardized (strictly RC-AVF) population of 945 patients, these systemic biomarkers lack independent clinical significance in predicting AVF thrombosis or 1-year patency loss when adjusted for local anatomical parameters.

Current literature contains numerous studies that contradict our findings, claiming that systemic inflammatory indices strongly predict AVF failure. One study reported that high preoperative NLR, PLR, and SII values were directly associated with AVF maturation failure and early thrombosis [7]. Similarly, publications argue that preoperative NLR (>2.7) significantly predicts early AVF failure [8], and that SII is an independent risk factor for distal AVF failure [9]. Furthermore, predictive models have been developed claiming that SII holds the strongest prognostic value for vascular access survival [10], suggesting that high inflammation indices reduce AVF blood flow and exhibit a sex-specific relationship with inadequate maturation [11].

However, the methodological limitations of these studies should not be overlooked. Many of these publications involve relatively small sample sizes and analyses prone to “overfitting,” where specific anatomical and hemodynamic parameters are not sufficiently integrated into the statistical models. In our cohort of 945 patients, which possesses high statistical power, no significant difference was found regarding any inflammatory markers between patients with patent fistulas and those with thrombosed ones (*p*-values > 0.25 for all). Moreover, even in high-risk subgroups with severe endothelial dysfunction such as Diabetes, CAD, or PAD, the AUC values for these indices remained clinically uninformative, failing to establish a reliable prognostic threshold. Rather than viewing previous estimates as merely exaggerated, an alternative interpretation is that inflammatory and nutritional biomarkers may have context-dependent value; they might contribute incrementally to broader prognostic models or predict overall mortality but fail to independently predict localized AVF survival when dominant anatomical parameters are accounted for.

Attempting to predict mortality is not equivalent to predicting fistula dysfunction. The nuanced role of systemic inflammation in vascular surgery has been recently elucidated by Scalise et al. [14] in their comprehensive review of inflammation biomarkers in carotid artery interventions. Their conceptual framework emphasizes that while systemic inflammation is mechanistically relevant, the predictive performance of biomarkers varies significantly depending on the specific vascular bed, local remodeling processes, procedural characteristics, and chosen endpoints [14]. Our findings align with this perspective; while inflammation and nutritional loss undeniably impact the overall “Malnutrition–Inflammation–Atherosclerosis (MIA) Syndrome” and premature aging in chronic kidney disease patients [4,5], and nutritional indices like PNI and GNRI have proven highly valuable in estimating general mortality and frailty in massive cohorts of over 100,000 patients [12]. Similarly, there are studies demonstrating that NLR and PLR predict cardiovascular mortality [13,15]. While these findings are clinically accurate regarding overall survival, the critical distinction presented by our study is this: the ability of an index to predict a patient’s overall mortality or systemic survival does not mean that index can reliably predict the localized thrombosis of a surgical anastomosis in the arm.

The failure of systemic inflammatory indices to predict AVF outcomes in our cohort supports evidence that seeks the solution in the localized pathophysiological response of the vessel wall rather than in systemic blood values. AVF maturation is an adaptive biomechanical response of endothelial cells to locally increased blood flow and shear stress [3,16]. Even when patients have high systemic inflammatory cytokine profiles [17], venous arterialization occurs successfully if the surgical anastomosis is flawless and local hemodynamics are adequate. It has been clearly shown that the primary determinants in vascular remodeling are the quality of laminar flow and local endothelial adaptation [18,19]. Literature supports that small preoperative vessel diameters are the strongest predictors of AVF failure [20], and fistulas created below clinical guideline recommendations (<2 mm vein) yield poor results [21,22]. Furthermore, the predictive power of anatomical criteria evaluated by preoperative Doppler ultrasonography, known as the ‘Rule of 6s’ (blood flow volume > 600 mL/min, vein depth < 6 mm, and diameter > 6 mm), consistently shows high accuracy with a Positive Predictive Value (PPV) exceeding 90% and AUC scores approaching 0.78 [23]. In our study, multivariate logistic regression (Table 6) definitively confirmed this established paradigm: preoperative arterial and venous diameters emerged as the absolute strongest independent predictors of success, whereas systemic biomarkers provided no additional prognostic value.

Our findings convey a critical message to the vascular surgery community: this study serves as a recalibration of current evidence. While systemic inflammatory indices should not be categorically dismissed as uninformative in broader cardiovascular care, the clinical utility of routine preoperative composite blood indices (PNI, GNRI, NLR, PLR, SII) in predicting localized AVF patency may be significantly exaggerated in current literature. Vascular surgeons should approach biomarker-driven vascular access planning with caution. Instead of relying solely on systemic composite indices, patient-specific clinical risk factors and precise local anatomical and hemodynamic assessments should be meticulously analyzed [24].

### Study Limitations

This study has several methodological limitations. First, our study has a retrospective and single-center design, which may inherently lead to potential selection bias. However, our large and standardized (100% RC-AVF) sample size of 945 patients significantly mitigates this effect. Second, all analyzed nutritional and inflammatory composite indices (PNI, GNRI, NLR, PLR, SII) were calculated from laboratory data at a single preoperative time point. Since systemic inflammation is a highly dynamic process in hemodialysis patients, the lack of analysis regarding postoperative early or late fluctuations is a limitation. Third, while our multivariate model successfully adjusted for gross anatomical vessel diameters and baseline demographics, residual confounding from unmeasured variables cannot be entirely excluded. Finally, molecular changes in intraoperative specific shear stress and intravascular flow dynamics, which are among the most important physiological determinants of localized AVF thrombosis, could not be evaluated due to the retrospective nature of our study. Future multicenter and prospective studies integrating these indices with hemodynamic parameters will further reinforce our findings.

## 5. Conclusions

In conclusion, data from our large cohort of 945 patients definitively demonstrate that composite nutritional and inflammatory indices (PNI, GNRI, NLR, PLR, and SII) calculated from routine preoperative blood parameters hold no significant independent value in predicting autologous AVF maturation or 1-year primary patency when adjusted for local anatomy. Our study shows that even if systemic inflammatory load or nutritional status can reflect overall mortality, it cannot reliably determine the thrombotic fate of a localized vascular anastomosis. Therefore, vascular surgeons should re-evaluate their reliance on these systemic indices and refocus surgical planning on meticulous preoperative Doppler ultrasonography mapping, optimization of surgical technique, and precise local hemodynamic assessments.

## Figures and Tables

**Table 1:** Baseline demographic characteristics, comorbidities, and surgical vessel details of the study cohort. (Note: Continuous variables are expressed as Mean ± Standard Deviation (SD), and categorical variables as number (*n*) and percentage (%). CAD: Coronary Artery Disease, PAD: Peripheral Artery Disease, AVF: Arteriovenous Fistula.).

Characteristic	Value (*n* = 945)
Age (Years, Mean ± SD)	61.5 ± 11.6
Gender (Male, %)	55.0% (*n* = 520)
Comorbidities (%)	
- Hypertension	79.0% (*n* = 747)
- Diabetes Mellitus	44.7% (*n* = 422)
- Coronary Artery Disease (CAD)	33.4% (*n* = 316)
- Peripheral Artery Disease (PAD)	16.5% (*n* = 156)
Preoperative Vessel Diameters (mm)	
- Arterial Diameter (Mean ± SD)	3.24 ± 0.90
- Venous Diameter (Mean ± SD)	3.62 ± 1.04
Surgical AVF Type (%)	
- Radio-cephalic (RC-AVF)	100.0% (*n* = 945)
- Brachio-cephalicor Brachio-basilic	Excluded

**Table 2:** Clinical Outcomes: AVF Maturation and Patency Rates.

Outcome Measure	Rate/Number
Maturation Success Rate	79.8%
1-Year Primary Patency	73.3% (*n* = 693)
1-Year Primary Failure/Thrombosis	26.7% (*n* = 252)
1-Year Secondary Patency	93.1% (*n* = 880)

**Table 3:** Comparison of preoperative systemic indices between patent and failed (thrombosed) AVF groups at 1-year follow-up. (Note: No statistically significant differences were observed between the groups; all *p*-values > 0.05. Independent samples *t*-test was utilized.).

Biomarker (Index)	Patent Group (*n* = 693)	Failed Group (*n* = 252)	*p* Value
SII (Systemic Immune-Inflammation)	885.00	861.37	0.412
NLR (Neutrophil-to-Lymphocyte)	3.48	3.37	0.355
PLR (Platelet-to-Lymphocyte)	170.40	169.79	0.824
PNI (Prognostic Nutritional)	45.85	45.74	0.751
GNRI (Geriatric Nutritional)	103.73	103.00	0.688

**Table 4:** Diagnostic performance of preoperative composite inflammatory and nutritional indices in predicting 1-year AVF patency loss (ROC Curve Analysis). (Note: An Area Under the Curve (AUC) of 0.50 indicates no discrimination. None of the composite indices demonstrated a statistically significant predictive value, all *p* > 0.05. CI: Confidence Interval).

Index	Area Under Curve (AUC)	95% Confidence Interval (CI)	*p* Value
GNRI	0.518	0.475–0.561	0.399
PNI	0.502	0.459–0.545	0.925
PLR	0.490	0.447–0.533	0.637
NLR	0.479	0.436–0.522	0.319
SII	0.476	0.433–0.519	0.254

**Table 5:** Predictive value of systemic indices for AVF failure across high-risk subgroups (Diabetes Mellitus, Coronary Artery Disease, Peripheral Artery Disease) (*p*-values). (Note: In none of these high-risk subgroups with severe endothelial dysfunction did inflammatory or nutritional indices predict AVF thrombosis; *p* > 0.05 in all analyses.).

Subgroup	Sample (*n*)	SII (*p*)	NLR (*p*)	GNRI (*p*)	PNI (*p*)
Diabetes Mellitus	422	0.40	0.31	0.60	0.06
Coronary Artery Dis.	316	>0.60	>0.60	>0.60	>0.60
Peripheral Artery Dis.	156	>0.14	>0.14	>0.14	>0.14

**Table 6:** Multivariate Logistic Regression Analysis for Predictors of 1-Year AVF Failure. (Note: * Indicates a statistically significant independent predictor, *p* < 0.05. An Odds Ratio < 1 indicates a protective effect against 1-year AVF failure).

Variable	Odds Ratio (OR)	95% CI	*p* Value
Age (per year)	1.01	0.99–1.03	0.215
Gender (Male vs. Female)	0.95	0.70–1.28	0.742
Diabetes Mellitus	1.25	0.91–1.72	0.168
Preop. Arterial Diameter (per 1 mm)	0.58	0.46–0.73	<0.001 *
Preop. Venous Diameter (per 1 mm)	0.51	0.41–0.63	<0.001 *
SII (Systemic Immune-Inflammation)	1.00	0.99–1.01	0.855
NLR (Neutrophil-to-Lymphocyte)	1.02	0.94–1.11	0.640
PNI (Prognostic Nutritional)	0.98	0.93–1.04	0.512

## Data Availability

The data presented in this study are available on request from the corresponding author due to privacy and ethical restrictions.

## References

[1] Vascular Access Work Group (2006). Clinical practice guidelines for vascular access. Am. J. Kidney Dis..

[2] Allon M., Robbin M.L. (2002). Increasing arteriovenous fistulas in hemodialysis patients: Problems and solutions. Kidney Int..

[3] Brahmbhatt A., Remuzzi A., Franzoni M., Misra S. (2016). The molecular mechanisms of hemodialysis vascular access failure. Kidney Int..

[4] Cobo G., Lindholm B., Stenvinkel P. (2018). Chronic inflammation in end-stage renal disease and dialysis. Nephrol. Dial. Transplant..

[5] Ebert T., Pawelzik S.C., Witasp A., Arefin S., Hobson S., Kublickiene K., Shiels P.G., Bäck M., Stenvinkel P. (2020). Inflammation and premature ageing in chronic kidney disease. Toxins.

[6] Kadatane S.P., Satariano M., Massey M., Mongan K., Raina R. (2023). The role of inflammation in CKD. Cells.

[7] Kaller R., Arbănași E.M., Mureșan A.V., Voidăzan S., Arbănași E.M., Horváth E., Suciu B.A., Hosu I., Halmaciu I., Brinzaniuc K. (2022). The predictive value of systemic inflammatory markers, the prognostic nutritional index, and measured vessels’ diameters in arteriovenous fistula maturation failure. Life.

[8] Wongmahisorn Y. (2019). Role of neutrophil-to-lymphocyte ratio as a prognostic indicator for hemodialysis arteriovenous fistula failure. J. Vasc. Access.

[9] Bayici B.B., Kizilyel F. (2026). Preoperative systemic immune-inflammation index is independently associated with distal arteriovenous fistula failure. Ann. Vasc. Surg..

[10] Ren S., Xv C., Wang D., Xiao Y., Yu P., Tang D., Yang J., Meng X., Zhang T., Zhang Y. (2024). The predictive value of systemic immune-inflammation index for vascular access survival in chronic hemodialysis patients. Front. Immunol..

[11] Akkaya Ö., Arslan Ü. (2025). Sex-specific impact of inflammation and nutritional indices on AVF blood flow and maturation: A retrospective analysis. Diagnostics.

[12] Miyasato Y., Hanna R.M., Morinaga J., Mukoyama M., Kalantar-Zadeh K. (2023). Prognostic nutritional index as a predictor of mortality in 101,616 patients undergoing hemodialysis. Nutrients.

[13] Barutcu Atas D., Tugcu M., Asicioglu E., Velioglu A., Arikan H., Koc M., Tuglular S. (2022). Prognostic nutritional index is a predictor of mortality in elderly patients with chronic kidney disease. Int. Urol. Nephrol..

[14] Scalise E., Costa D., Bolboacă S.D., Ielapi N., Bevacqua E., Cristodoro L., Faga T., Michael A., Andreucci M.V., Bracale U.M. (2025). The role of inflammation biomarkers in carotid artery stenosis procedures. Ann. Vasc. Surg..

[15] Zhang J., Lu X., Wang S., Li H. (2021). High neutrophil-to-lymphocyte ratio and platelet-to-lymphocyte ratio are associated with poor survival in patients with hemodialysis. Biomed. Res. Int..

[16] Remuzzi A., Bozzetto M. (2017). Biological and physical factors involved in the maturation of arteriovenous fistula for hemodialysis. Cardiovasc. Eng. Technol..

[17] Martinez L., Perla M., Tabbara M., Duque J.C., Rojas M.G., Falcon N.S., Pereira-Simon S., Salman L.H., Vazquez-Padron R.I. (2022). Systemic profile of cytokines in arteriovenous fistula patients and their associations with maturation failure. Kidney360.

[18] Kudze T., Ono S., Fereydooni A., Gonzalez L., Isaji T., Hu H., Yatsula B., Taniguchi R., Koizumi J., Nishibe T. (2020). Altered hemodynamics during arteriovenous fistula remodeling leads to reduced fistula patency in female mice. JVS Vasc. Sci..

[19] Chan S.M., Weininger G., Langford J., Jane-Wit D., Dardik A. (2021). Sex differences in inflammation during venous remodeling of arteriovenous fistulae. Front. Cardiovasc. Med..

[20] Waheed A., Masengu A., Skala T., Li G., Jastrzebski J., Zalunardo N. (2020). A prospective cohort study of predictors of upper extremity arteriovenous fistula maturation. J. Vasc. Access.

[21] van Vliet L.V., Zonnebeld N., Tordoir J.H., Huberts W., Bouwman L.H., Cuypers P.W., Heinen S.G., Huisman L.C., Lemson S., Mees B.M. (2024). Guideline recommendations on minimal blood vessel diameters and arteriovenous fistula outcomes. J. Vasc. Access.

[22] He Y., Northrup H., Roy-Chaudhury P., Cheung A.K., Berceli S.A., Shiu Y.T. (2021). Analyses of hemodialysis arteriovenous fistula geometric configuration and its associations with maturation and reintervention. J. Vasc. Surg..

[23] (2006). Hemodialysis Adequacy 2006 Work Group. Clinical practice guidelines for hemodialysis adequacy, update 2006. Am. J. Kidney Dis..

[24] See Y.P., Cho Y., Pascoe E.M., Cass A., Irish A., Voss D., Polkinghorne K.R., Hooi L.S., Ong L.M., Paul-Brent P.A. (2020). Predictors of arteriovenous fistula failure: A post hoc analysis of the FAVOURED study. Kidney360.

